# Detection of Hepatitis E Virus in *Hyalomma lusitanicum* Ticks Feeding on Wild Boars

**DOI:** 10.3389/fmicb.2021.692147

**Published:** 2021-07-09

**Authors:** Antonio Rivero-Juarez, María A. Risalde, Christian Gortázar, Pedro Lopez-Lopez, Jose A. Barasona, Mario Frias, Javier Caballero-Gomez, José de la Fuente, Antonio Rivero

**Affiliations:** ^1^Grupo de Virología Clínica y Zoonosis, Unidad de Enfermedades Infecciosas, Instituto Maimónides de Investigación Biomédica de Córdoba, Hospital Reina Sofía, Universidad de Córdoba, Córdoba, Spain; ^2^Departamento de Anatomía y Anatomía Patológica Comparadas y Toxicología, Facultad de Veterinaria, Universidad de Córdoba, Córdoba, Spain; ^3^Sanidad y Biotecnología, Instituto de Investigación en Recursos Cinegéticos (UCLM-CSIC-JCCM), Ciudad Real, Spain; ^4^VISAVET, Animal Health Department, Veterinary School, Complutense University of Madrid, Madrid, Spain; ^5^Departamento de Sanidad Animal, Facultad de Veterinaria, Universidad de Córdoba, Córdoba, Spain; ^6^Department of Veterinary Pathobiology, Center for Veterinary Health Sciences, Oklahoma State University, Stillwater, OK, United States

**Keywords:** hepatitis E, *Hyalomma lusitanicum*, tick, epidemiology, wild boar

## Abstract

Little is known about the role of ticks in maintaining highly prevalent zoonotic viruses in wildlife, such as hepatitis E virus (HEV), which do not require ticks for transmission between animals and humans. In this cross-sectional study, adult female ticks were collected from Eurasian wild boar (*Sus scrofa*) in autumn 2015 in Spain. HEV RNA in both ticks and wild boar was evaluated by RT-qPCR. Twenty-nine adult *Hyalomma lusitanicum* ticks were collected from 29 wild boars. HEV RNA was detected in a total of 10 tick (34.4%) and 11 wild boar serum samples (37.9%). In two cases, detectable HEV RNA was found in a wild boar but not in the tick collected from them. In contrast, one HEV-positive tick was collected from an HEV-negative wild boar. All viral sequences were consistent with genotype 3f. We describe for the first time the presence of HEV RNA in adult *Hyalomma lusitanicum* ticks.

## Introduction

The role of ticks in the maintenance and spread of emerging viral zoonotic pathogens, whose main hosts are wildlife species, is well known (Ruiz-Fons et al., 2008; Baneth, 2014; Madison-Antenucci et al., 2020). Of special concern are wild ungulates (deer and wild swine), species with wide distribution and high density, which allow the spread of tick-borne viruses in Europe (Kriz et al., 2014). In this sense, viruses belonging to families *Flaviviridae* (tick-borne encephalitis), *Nairoviridae* (Crimean–Congo hemorrhagic fever), and *Phenuiviridae* (severe fever with thrombocytopenia) have emerged in several European countries (Madison-Antenucci et al., 2020), such as Spain and the Czechia (Kriz et al., 2014; Moraga-Fernández et al., 2020), spread by wild boar. Nevertheless, little is known about the role of ticks in maintaining other highly prevalent zoonotic viruses in wildlife, such as hepatitis E virus (HEV), which do not require ticks for transmission between animals and humans.

Hepatitis E virus genotypes 3 and 4 have been detected in a great variety of domestic and wild mammals, with suids considered as the main host (Wang and Meng, 2021), and the main transmission route being the consumption of raw or undercooked meat or organs (Faber et al., 2018). Feral ungulates, principally wild boar (*Sus scrofa*), constitute the main wild reservoir of the virus, with a reported prevalence of infection of 20% (Rivero-Juarez et al., 2018), which means that wild boar meat consumption is an important route of transmission in Europe (Faber et al., 2018). Even though the main route of HEV transmission in wild boar is not well understood, it has been suggested that it could be related to direct contact between the animals and other species, including other sympatric species (deer and fallow deer) and pigs, or by indirect contact spread through feces (Rivero-Juarez et al., 2018). In contrast to other highly prevalent viruses in wild boar, there are no studies evaluating the presence of HEV in ticks feeding on this species. Information on this point is of great interest with respect to increasing knowledge about HEV epidemiology.

## Materials and Methods

### Wild Boar Sampling and Collection

We designed a study in which adult female ticks were collected from Eurasian wild boar (*S. scrofa*) sampled from hunting estates in southern Spain between October and November 2015. The climate in this area is semi-arid Mediterranean with continental influences, a pronounced dry season, and annual rainfall of 400 to 500 mm with high inter-annual variability. The dry season (from June to September, with considerable annual variations typical of the Mediterranean climate) means that there is a period in which there is a shortage of food and water. The wet season typically starts in September–October, lasts until spring, and contributes most to the annual rainfall. The altitude ranges between 190 and 452 m ASL. The habitat is characterized as *dehesa*, with evergreen oak forests dominated by *Quercus* sp. and scrublands (*Cystus* sp., *Pistacia* sp., *Rosmarinus* sp., *Erica* sp., and *Phyllirea* sp.), scattered pastures, and small crops (“dehesas”). The possibility of contact with other domestic species on hunting estates is extremely low since these are fenced estates. There may be sporadic cross-fence contact at the boundaries: with sheep in the western area and cattle in the northern area. Other common wildlife species of the Mediterranean habitat coexist with wild boar on these hunting estates. Among mammals, red deer are especially abundant, and the European wild rabbit, red fox, and Iberian hare are present in low numbers. These hunting estates are intensively managed, with supplementary feeding throughout the year and perimeter fencing. The wild boar density in the area is approximately 5–10 wild boar/km^2^. The average number of wild boar hunted per year on estates is 80.8, mainly adults, with a balanced sex ratio. For the purpose of the study, the hunted wild boars for that year were randomly selected, and those with ticks were included in the study.

A whole blood sample was obtained from all hunted wild boars by puncture and transported under refrigerated conditions to the laboratory, where serum was obtained after centrifugation of the whole blood at 10,000 rpm for 10 min. Serum was submerged in RNAlater^®^ Stabilization Solution (Thermo Fisher Scientific Inc., Waltham, MA, United States) and frozen at −80°C until RNA extraction. Viral RNA was extracted from 200 μl of serum with a commercial QIAamp MinElute Virus Spin Kit (Qiagen, Hilden, Germany), by an automated procedure (QIAcube. Qiagen, Hilden, Germany).

### Tick Sampling and Collection

Ticks were collected from the surface of the wild boar sampled. Tick transportation, handling, and identification were performed in accordance with previously described procedures (Moraga-Fernández et al., 2020). Briefly, the ticks were kept alive in labeled sterile vials and transported at room temperature with controlled humidity to the laboratory, where they were identified using taxonomic keys (Walker et al., 2004) and stored at −80°C. Molecular identification, amplifying a mitochondrial 16S rDNA target of 460 bp, was then performed in order to confirm the previous morphologic identification. RNA was extracted from the whole tick using the RNeasy Mini Kit (Qiagen, Hilden, Germany), using the same automated procedure as in serum samples.

### HEV Molecular Evaluation and Sequencing

Hepatitis E virus RNA was evaluated by RT-qPCR using a protocol developed and validated by our group targeting the ORF3 region (Frías et al., 2021). The detection limit of this assay was set at 21 IU/ml. Those samples exhibiting detectable viral load were genotyped by nested RT-PCR targeting a 420-bp genome fragment located in the ORF2 region, using a protocol reported previously (Frías et al., 2021). The PCR product was sequenced using the BigDye Terminator Cycle Sequencing Ready Reaction Kit on an ABI PRISM 3100 Genetic Analyzer (Applied Biosystems, Foster City, CA, United States). The consensus sequence was obtained using SeqMan NGen^®^ software version 12.0 (DNASTAR. Madison, WI, United States). Subtype assignment and phylogenetic analyses were performed using the *HEVnet* genotyping tool^1^ (Mulder et al., 2019). Sequence alignments were generated by the MAFFT online service, and phylogenetic trees were constructed using the maximum likelihood method and the recently proposed HEV genotype/subtype standard reference (Smith et al., 2020). The final tree was obtained with MEGA Software (version 6) using the bootstrap method (bootstrapped with 1,000 replicates). *P*-distances were calculated and compared for wild boar and tick sequences.

### Ethical Approval

This study did not involve purposeful killing of animals. All samples were collected from legally hunted animals during the hunting season or by passive surveillance under Spanish and Andalusian legislation. No ethical approval was necessary.

## Results

A total of 29 adult ticks, all identified as *Hyalomma lusitanicum* (Koch,1844), were collected from 29 wild boars hunted during the study period. With respect to the wild boar, 12 females and 17 males were analyzed, 19 of which were adults, 6 sub-adults, and 4 juveniles. Age was determined on the basis of tooth eruption; animals that were under 12 months old were classified as juveniles, those between 12 and 24 months as sub-adults, and those over 2 years old as adults. All wild boars had only one tick. HEV RNA was detected in a total of 10 ticks (34.5%) and 11 wild boars (37.9%) (Table 1). In two cases, detectable HEV RNA was found in a wild boar but not in the tick collected from them. In contrast, one HEV-positive tick was collected from an HEV-negative wild boar (Table 1).

**TABLE 1:** Distribution of hepatitis E-positive wild boars (*Sus scrofa*) and ticks (*Hyalomma lusitanicum*).

**Wild boar ID**	**HEV (IU/ml)**	**Genotype (ID)**	**Tick ID**	**HEV (Ct)**	**Genotype (ID)**
J-1695	Positive (6,954)	3f (MT822891)	G-004	Negative	–
J-1696	Positive (6,762)	3f (MT822892)	G-005	Negative	–
J-1701	Positive (145,643)	3f (MT822888)	G-010	Positive (37.7)	Not sequenced
J-1488	Positive (3,385)	Not sequenced	G-954	Positive (33.1)	3f (MW074086)
J-1490	Positive (42,956)	3f (MT822887)	G-956	Positive (30.2)	3f (MW074087)
J-1491	Positive (24,314)	3f (MT822893)	G-957	Positive (31.4)	3f (MW074088)
J-1493	Positive (2,926)	Not sequenced	G-959	Positive (30.3)	3f (MW074089)
J-1495	Positive (266,698)	3f (MT822889)	G-962	Positive (27.6)	3f (MW074090)
J-1499	Positive (1,763)	Not sequenced	G-966	Positive (28.1)	3f (MW074091)
J-1501	Positive (1,032)	Not sequenced	G-967	Positive (30.1)	3f (MW074092)
J-1502	Positive (264,752)	3f (MT822895)	G-968	Positive (34.1)	3f (MW074093)
J-1505	Negative	–	G-970	Positive (31.7)	3f (MW074094)

*ID, animal identification number; HEV, hepatitis E virus.*

**FIGURE 1:**
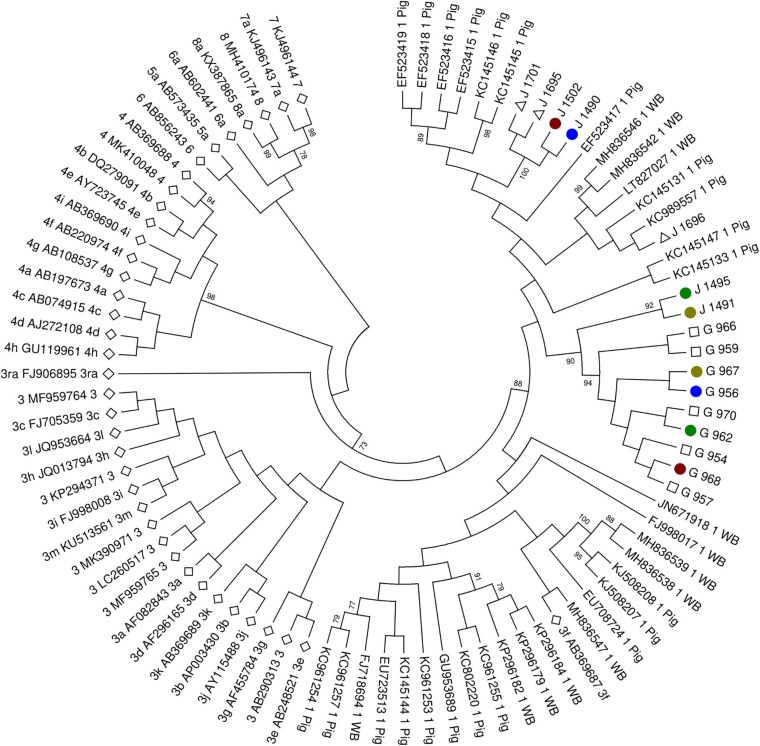
Molecular phylogenetic analysis by maximum likelihood method. White diamond highlights the standard strains proposed by Smith et al. (2020). White triangle highlights wild boar sequences without positive ticks for hepatitis E virus (HEV) RNA. White square highlights tick sequences without positive wild boar for HEV RNA. Colored circles highlight pair wild boar–tick sequences (red for J1502-G968, blue for J1490-G956, dark green for J1495-G962, and light green for J1491-G967). The evolutionary history was inferred by using the maximum likelihood method based on the Tamura-Nei model. The bootstrap consensus tree inferred from 1,500 replicates is taken to represent the evolutionary history of the taxa analyzed. Branches corresponding to partitions reproduced in less than 50% bootstrap replicates are collapsed. Initial tree(s) for the heuristic search were obtained by applying the neighbor-joining method to a matrix of pairwise distances estimated using the maximum composite likelihood (MCL) approach. The analysis involved 88 nucleotide sequences. All positions with less than 95% site coverage were eliminated. That is, fewer than 5% alignment gaps, missing data, and ambiguous bases were allowed at any position. There was a total of 302 positions in the final dataset. Evolutionary analyses were conducted in MEGA6.

Of the 21 positive samples, 16 samples could be sequenced (seven wild boar samples and nine tick samples). All sequences were consistent with genotype 3f (Figure 1), showing a high homology between them. Three samples (two wild boars and one tick) could not be sequenced because of low HEV RNA titer (Ct value higher than 35). The mean distance between wild boar sequences was 0.05 and between tick sequences, 0.001. The mean distance between groups (wild boar and ticks) was 0.06.

## Discussion

Our study detected HEV RNA in ticks feeding on wild boar, providing, to our knowledge, the first identification of this virus in ticks. We found that in 9 out of 11 (81.8%) of the wild boars with detectable HEV viral load, an HEV-positive tick was also detected. This finding suggests that the positivity of ticks could be related to recent blood meals from the animal from which they were collected. Consequently, the detection of HEV-positive ticks in our study could be regarded as merely an indirect marker of positivity in the host. The identification of an HEV-positive tick collected from an HEV-negative wild boar, however, could imply that ticks could acquire HEV by feeding from other animals; this result leads us to hypothesize that transmission of HEV between animals through the bites of virus-carrying ticks could occur.

This potential association has been suggested for other non-tick-borne viruses that are highly prevalent in wild boars (Franzo et al., 2019). Porcine circovirus 3 (PCV3) was recently isolated from ticks (*Ixodes ricinus*) feeding on virus-negative wild boars and deer (Franzo et al., 2019). The authors hypothesized that ticks could play a role in the transmission and maintenance of PCV3 in the sylvatic interface. Our finding does not allow us to argue in favor of HEV transmission through ticks. To make this assertion, other experimental approaches are necessary, including identification of the virus in unfed ticks and salivary glands, and an evaluation of transmission efficiency using animal models that include key aspects such as infectious dose and amount of virus present in the tick, the viability of the virus in the tick, and time between two blood meals on two different wild boars and survival of the virus. Nevertheless, our results support the initiation of studies to evaluate whether HEV can be transmitted through tick bites. Although consumption of raw or undercooked meat will remain the main transmission route of zoonotic HEV infection (Faber et al., 2018), the possibility of maintenance and transmission of HEV through ticks is interesting and needs to be evaluated. If further results confirm the efficiency of HEV transmission by this route, such transmission could facilitate the sympatric spread of HEV from wild boar to other species, as has been demonstrated for other viruses (Kriz et al., 2014; Faber et al., 2018; Negredo et al., 2019). Consequently, the role of ticks in the epidemiology of HEV requires further investigation.

The HEV prevalence detected in our study could be considered high. There are two possible explanations for this finding. Firstly, the prevalence reported here is consistent with other studies conducted in our setting. In the majority of studies conducted in Spain, the prevalence of HEV infection found in this species is higher than 20%. In a study conducted by our group in the same area, the rate of infection in a population of 142 animals was 23.2% [95% confidence interval (CI): 16.8–30.7%] (Rivero-Juarez et al., 2018). Similarly, in a study conducted in northeastern Spain including 264 wild boars, the prevalence of infection was 20% (14.9–24.5) (Wang et al., 2019). Finally, in a study conducted in multiple sampling areas in south-central Spain including 150 wild boar, 19.6% (95% CI: 13.53–27.40%) were infected with HEV (de Deus et al., 2008). Furthermore, in this study, the HEV prevalence in different southern areas rose to over 40% and dropped to 7.7% in central areas (de Deus et al., 2008). This finding is consistent with another study in which the prevalence of viremic wild boar from central Spain was 10.12% (95% CI: 5.44–14.8) (Kukielka et al., 2016). Consequently, there is a clear variation in HEV prevalence in Spain depending on the hunting area, with the highest rate of infection being reported in south-central Spain, our sampling area. This association has also been reported in other European countries such as Germany, where the HEV prevalence was lower in the north (5.3%; 10 out of 189 animals) (Schielke et al., 2015) than in central Germany (15.2%; 7 out of 46) (Kaci et al., 2008), and rising to 68% in several areas (Adlhoch et al., 2009). The second explanation is that a seasonal pattern of HEV infection has been suggested for wild boar from our sampling area (Rivero-Juarez et al., 2018). Accordingly, the highest prevalence was reported in the first weeks of autumn (October–November), when more than 44% of the animals were infected. This prevalence decreased over time, with the percentage of infected animals dropping from 28% in December to just 1% in January and 0% in February. For the present study, samples were collected during the period with the highest number of infected animals (October–November). Consequently, a high prevalence was expected in our study.

## Conclusion

We describe for the first time the presence of HEV RNA in adult *H. lusitanicum* ticks. Studies evaluating the role of ticks in HEV epidemiology are warranted.

## Data Availability Statement

All data generated or analyzed during the study are included in this published article. The datasets used and/or analyzed during the present research project are available from the corresponding author on reasonable request. Sequences are available on GenBank under accession numbers: MT822891, MT822892, MT822888, MT822887, MT822893, MT822889, MT822895, MW074086, MW074087, MW074088, MW074089, MW074090, MW074091, MW074092, MW074093, and MW074094.

## Ethics Statement

Ethical review and approval was not required for the animal study because, all samples were collected from legally hunted animals during the hunting seasons or by passive surveillance under Spanish and Andalusian legislation. No ethical approval was necessary.

## Author Contributions

AR-J and MR designed the study. MR, CG, JB, and JF sampled the animals and ticks. AR-J, PL-L, MF, and J-CG gathered the molecular data. AR-J interpreted the data. AR-J and AR obtained funding and drafted the manuscript. All authors critically revised the draft for important intellectual content, contributed to the article, and approved the submitted version.

## Conflict of Interest

The authors declare that the research was conducted in the absence of any commercial or financial relationships that could be construed as a potential conflict of interest.

## Footnotes

1 https://www.rivm.nl/mpf/typingtool/hev/
